# Supplementary material for: Staphylococcus argenteus Infections, Brazil
Source: Microbiol Spectr. 2023 Jan 23;11(1):e01179-22. doi: 10.1128/spectrum.01179-22 (PMC9927369; doi:10.1128/spectrum.01179-22)
Supplement: Supplemental file 1 — Supplemental material. Download spectrum.01179-22-s0001.pdf, PDF file, 0.2 MB [file spectrum.01179-22-s0001.pdf]

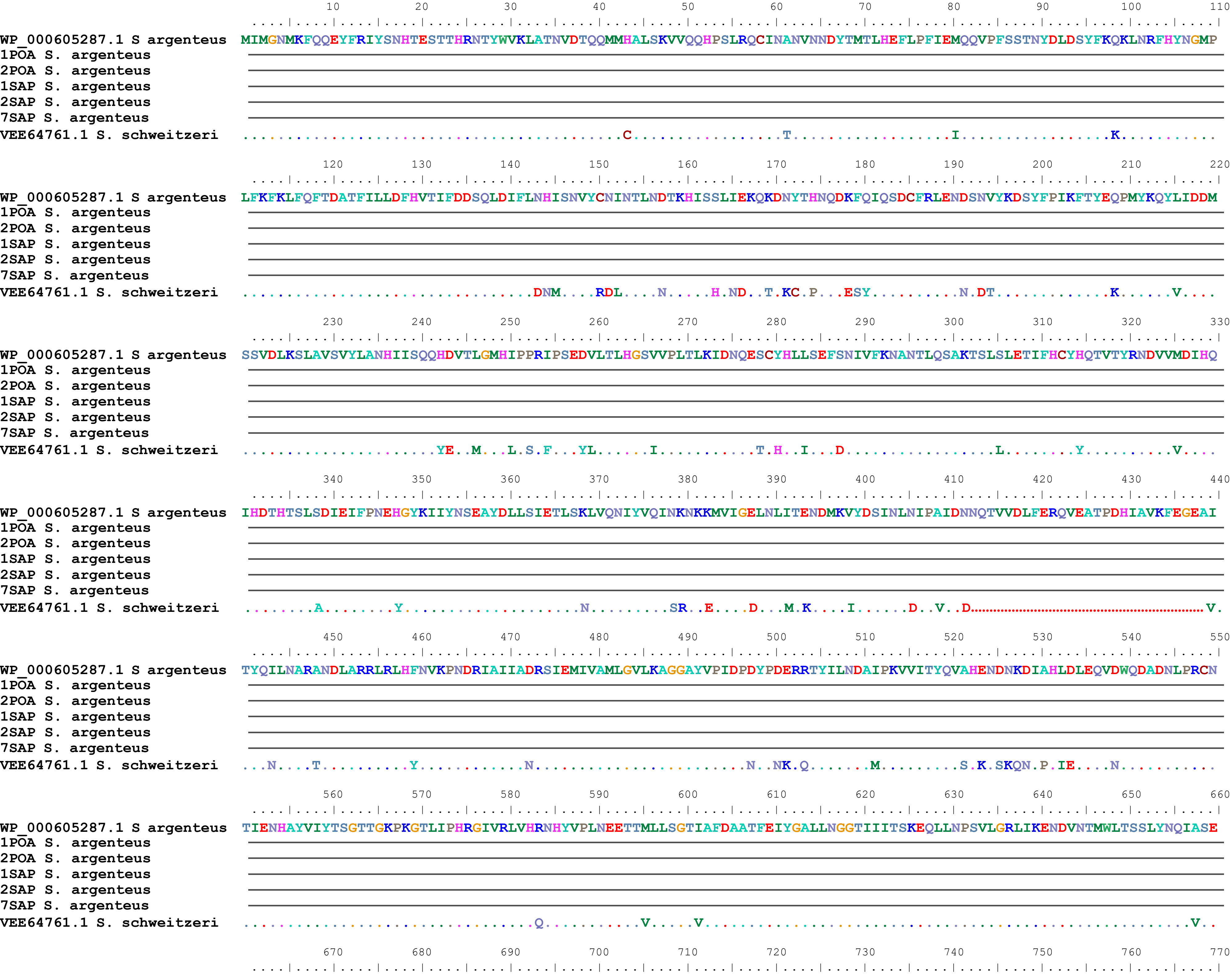

10 20 30 40 50 60 70 80 90 100 110
WP_000605287.1 S argenteus MIMGNMKFQQEYFRIYSNHTESTTHRNTYWVKLATNVDTQQMMHALSKVVQQHPSLRQCINANVNNDYTMTLHEFLPFIEMQQVPFSSTNYDLDSYFKQKLNRFHYNGMP
1POA S. argenteus
2POA S. argenteus
1SAP S. argenteus
2SAP S. argenteus
7SAP S. argenteus
VEE64761.1 S. schweitzeri
120 130 140 150 160 170 180 190 200 210 220
WP_000605287.1 S argenteus LFKFKLFQFTDATFILLDFHVTIFDDSQLDIFLNHISNVYCNINTLNDTKHISSLIEKQKDNYTHNQDKFQIQSDCFRLENDSNVYKDSYFPIKFTYEQPMYKQYLIDDM
1POA S. argenteus
2POA S. argenteus
1SAP S. argenteus
2SAP S. argenteus
7SAP S. argenteus
VEE64761.1 S. schweitzeri
230 240 250 260 270 280 290 300 310 320 330
WP_000605287.1 S argenteus SSVDLKSLAVSVYLANHIISQQHDVTLGMHIPPRIPSEDVLTLHGSVVPLTLKIDNQESCYHLLSEFSNIVFKNANTLQSAKTSLSLETIFHCYHQTVTYRNDVVMDIHQ
1POA S. argenteus
2POA S. argenteus
1SAP S. argenteus
2SAP S. argenteus
7SAP S. argenteus
VEE64761.1 S. schweitzeri
340 350 360 370 380 390 400 410 420 430 440
WP_000605287.1 S argenteus IHDTHTSLSDIEIFPNEHGYKIIYNSEAYDLLSIETLSKLVQNIYVQINKNKKMVIGELNLITENDMKVYDSINLNIPAIDNNQTVVDLFERQVEATPDHIAVKFEGEAI
1POA S. argenteus
2POA S. argenteus
1SAP S. argenteus
2SAP S. argenteus
7SAP S. argenteus
VEE64761.1 S. schweitzeri
450 460 470 480 490 500 510 520 530 540 550
WP_000605287.1 S argenteus TYQILNARANDLARRLRLHFNVKPNDRIAIIADRSIEMIVAMLGVLKAGGAYVPIDPDYPDERRTYILNDAIPKVVITYQVAHENDNKDIAHLDLEQVDWQDADNLPRCN
1POA S. argenteus
2POA S. argenteus
1SAP S. argenteus
2SAP S. argenteus
7SAP S. argenteus
VEE64761.1 S. schweitzeri
560 570 580 590 600 610 620 630 640 650 660
WP_000605287.1 S argenteus TIENHAYVIYTSGTTGKPKGTLIPHRGIVRLVHRNHYVPLNEETTMLLSGTIAFDAATFEIYGALLNGGTIIITSKEQLLNPSVLGRLIKENDVNTMWLTSSLYNQIASE
1POA S. argenteus
2POA S. argenteus
1SAP S. argenteus
2SAP S. argenteus
7SAP S. argenteus
VEE64761.1 S. schweitzeri
670 680 690 700 710 720 730 740 750 760 770

```
1POA S. argenteus
2POA S. argenteus
1SAP S. argenteus
2SAP S. argenteus
7SAP S. argenteus
VEE64761.1 S. schweitzeri    ......................VN..............................S......................Q....V........E..................

                                    780       790       800       810       820       830       840       850       860       870       880
                             ....|....|....|....|....|....|....|....|....|....|....|....|....|....|....|....|....|....|....|....|....|....|
WP_000605287.1 S argenteus   FIMNAKNHELMYKSGDIVRLLPDGNIDYLYRMDKQVKIRGFRIELSEIERALEHIQGINKAVVIVQTYDQEDYIVAYYEGMHILSNNKIKAQLRRTLPEYMIPVNFLRIK
1POA S. argenteus
2POA S. argenteus
1SAP S. argenteus
2SAP S. argenteus
7SAP S. argenteus
VEE64761.1 S. schweitzeri    ..V.....Q....................................................................................................E

                                    890       900       910       920       930       940       950       960       970       980       990
                             ....|....|....|....|....|....|....|....|....|....|....|....|....|....|....|....|....|....|....|....|....|....|
WP_000605287.1 S argenteus   QIPITMNGKIDKKSLPKMDFVDMGTYVPPSTDMEHILCQIFEDILNVQQVGIHDNFFELGGHSLKATLVVNHIEEYTSKRLKISDLLQKPTVFELAQAISTIQEQRYESI
1POA S. argenteus
2POA S. argenteus
1SAP S. argenteus
2SAP S. argenteus
7SAP S. argenteus
VEE64761.1 S. schweitzeri    .....I..........S...............I......................................R.....G................................

                                   1000      1010      1020      1030      1040      1050      1060      1070      1080      1090      1100
                             ....|....|....|....|....|....|....|....|....|....|....|....|....|....|....|....|....|....|....|....|....|....|
WP_000605287.1 S argenteus   PIAVNKEDYVLSSTQQRMYLLWQANPKDTVYNVPFLWRLSSDLNVSQLQRAIHQLIARHEILRTQYIVVDDEVRQRIKTDVEVDFEEVNTHFKDEQQIMRQYIEPFNLEK
1POA S. argenteus
2POA S. argenteus
1SAP S. argenteus
2SAP S. argenteus
7SAP S. argenteus
VEE64761.1 S. schweitzeri    ...M.........................................A..RH..........V.............................Y...................

                                   1110      1120      1130      1140      1150      1160      1170      1180      1190      1200      1210
                             ....|....|....|....|....|....|....|....|....|....|....|....|....|....|....|....|....|....|....|....|....|....|
WP_000605287.1 S argenteus   PSQIRFRYIRSPLHAYLFIDTHHIINDGMSNIQLVKDLNALYQNKKLSPLTLQYKDYSEWMAQRDMGRHRQYWISQFEDAVPILSMPTDFVRPNVKTTNGAMTSFSLSNT
1POA S. argenteus
2POA S. argenteus
1SAP S. argenteus
2SAP S. argenteus
7SAP S. argenteus
VEE64761.1 S. schweitzeri    .....V........S....................N....F....G................R................G.........Y....................

                                   1220      1230      1240      1250      1260      1270      1280      1290      1300      1310      1320
                             ....|....|....|....|....|....|....|....|....|....|....|....|....|....|....|....|....|....|....|....|....|....|
WP_000605287.1 S argenteus   TRRQLQQYVEKHQVTDFMFFMSVVMVLLGKYARKEDITVGSVMSARMHSDTERMLGMFANTLVYRGQPVKEKMWTQFLQEIKVHSLAAYEHQEYPFDCLVADLDQAHDAS
1POA S. argenteus
2POA S. argenteus
1SAP S. argenteus
2SAP S. argenteus
7SAP S. argenteus
VEE64761.1 S. schweitzeri    ..........E...............................................................A.......EL..........................

                                   1330      1340      1350      1360      1370      1380      1390      1400      1410      1420      1430
                             ....|....|....|....|....|....|....|....|....|....|....|....|....|....|....|....|....|....|....|....|....|....|
WP_000605287.1 S argenteus   RNPLFDVMLVLQNNETNHSHFGHSKLTHIPPKSVTSKFDLAFIIEEDCNNYTINIEYNSDLYRSQTIQYMGQQFIAMIEFILAHNAPLQIKDIPNDNTDLLDWVNTHVNN
1POA S. argenteus
2POA S. argenteus
```

```
2SAP S. argenteus            --------------------------------------------------------------------------------------------------------------
7SAP S. argenteus            --------------------------------------------------------------------------------------------------------------
VEE64761.1 S. schweitzeri    ...........................Y...............L....D...................N..........Y.....E................N.......

                                    1440      1450      1460      1470      1480      1490      1500      1510      1520      1530      1540
                             ....|....|....|....|....|....|....|....|....|....|....|....|....|....|....|....|....|....|....|....|....|....|
WP_000605287.1 S argenteus   RTLDVPGNKSIIEYFDEVAAQRSNHVALVMNDLTMSYETLQHYVNKIARILTSKGIGRGQRIALLVERSFEMIAAMLAAVKLGAVYVPIDTDCPKKRREIILEDAQIVAI
1POA S. argenteus            --------------------------------------------------------------------------------------------------------------
2POA S. argenteus            --------------------------------------------------------------------------------------------------------------
1SAP S. argenteus            --------------------------------------------------------------------------------------------------------------
2SAP S. argenteus            --------------------------------------------------------------------------------------------------------------
7SAP S. argenteus            --------------------------------------------------------------------------------------------------------------
VEE64761.1 S. schweitzeri    .................IV......A..............................V........A............T....VT.....I...................

                                    1550      1560      1570      1580      1590      1600      1610      1620      1630      1640      1650
                             ....|....|....|....|....|....|....|....|....|....|....|....|....|....|....|....|....|....|....|....|....|....|
WP_000605287.1 S.argenteus   MTYGVDIESTLPVIDLENVKCHHKPSKNGYGDRVNCNQDDETCTEGDDNQGCVEWVNSYDCTYDKALHNQYSHESDGVRDNQCEAVSDVQECETVDNHSCGEMMLDNEMY
1POA  S.argenteus            ---------..................Y................................................E.................................
2POA  S.argenteus            ---------..................Y................................................E.................................
1SAP  S.argenteus            ---------..................Y................................................E.................................
2SAP  S.argenteus            ---------..................Y................................................E.................................
7SAP  S.argenteus            ---------..................Y................................................E.................................
VEE64761.1 S.schweitzeri     .I.D....T.............Y...E.D.....S.G..Y..Y.K.N..H.R...A.Q.G.IS.E......D....EIL.....KM..I.G..IA..LR...........

                                    1660      1670      1680      1690      1700      1710      1720      1730      1740      1750      1760
                             ....|....|....|....|....|....|....|....|....|....|....|....|....|....|....|....|....|....|....|....|....|....|
WP_000605287.1 S argenteus   VIYTSGTTGVPKGVAVNQRNLLNLVYAWTKELQLEDTEVFMQHANIVFDASVMEIYCCLLNGHTLVIPNKEQRVDPVQLQQLISKNHITVASIPLQMCSMMGDFYIEKLI
1POA S. argenteus            --------------------------------------------------------------------------------------------------------------
2POA S. argenteus            --------------------------------------------------------------------------------------------------------------
1SAP S. argenteus            --------------------------------------------------------------------------------------------------------------
2SAP S. argenteus            --------------------------------------------------------------------------------------------------------------
7SAP S. argenteus            --------------------------------------------------------------------------------------------------------------
VEE64761.1 S. schweitzeri    A.............V.............SH....Q.....L...............S.............A.....E......T.H...............K........

                                    1770      1780      1790      1800      1810      1820      1830      1840      1850      1860      1870
                             ....|....|....|....|....|....|....|....|....|....|....|....|....|....|....|....|....|....|....|....|....|....|
WP_000605287.1 S argenteus   TGGATSTPSFVNYIEHHCGTYYNAYGPSESTVITSYWSHQCADKVPETIPIGKPLSNIQVYIMDDGLLCGVGMPGELCIAGDSLTLGYINRPELTADKWQDNPFGKGKLY
1POA S. argenteus            --------------------------------------------------------------------------------------------------------------
2POA S. argenteus            --------------------------------------------------------------------------------------------------------------
1SAP S. argenteus            --------------------------------------------------------------------------------------------------------------
2SAP S. argenteus            --------------------------------------------------------------------------------------------------------------
7SAP S. argenteus            --------------------------------------------------------------------------------------------------------------
VEE64761.1 S. schweitzeri    ..............................................K................S....................A.....C....D..............

                                    1880      1890      1900      1910      1920      1930      1940      1950      1960      1970      1980
                             ....|....|....|....|....|....|....|....|....|....|....|....|....|....|....|....|....|....|....|....|....|....|
WP_000605287.1 S argenteus   HSGDLARYTPDGQIEFLGRIDKQVKVNGYRIELDEIENAMRAIHGVSDCVVTVNHFDTHDILNAYYVGQPHIEQHVKHHLLEHLPKYMVPKTVTHMNQMPLTANDKVDKS
1POA S. argenteus            --------------------------------------------------------------------------------------------------------------
2POA S. argenteus            --------------------------------------------------------------------------------------------------------------
1SAP S. argenteus            --------------------------------------------------------------------------------------------------------------
2SAP S. argenteus            --------------------------------------------------------------------------------------------------------------
7SAP S. argenteus            --------------------------------------------------------------------------------------------------------------
VEE64761.1 S. schweitzeri    ........................................L............K.......................Y.................I.H............

                                    1990      2000      2010      2020      2030      2040      2050      2060      2070      2080      2090
                             ....|....|....|....|....|....|....|....|....|....|....|....|....|....|....|....|....|....|....|....|....|....|
WP_000605287.1 S argenteus   QLPEPSILQQNGKMDSEATNEIEQTFINVFETVLKQNHIGVDDDFFELGGNSLEAMLVVSRLKVFGHHISMQTLYQYKTVKRIVKYMHQCQQLEVMLPKDYPELQQMVES
1POA S. argenteus            --------------------------------------------------------------------------------------------------------------
2POA S. argenteus            --------------------------------------------------------------------------------------------------------------
1SAP S. argenteus            --------------------------------------------------------------------------------------------------------------
2SAP S. argenteus            --------------------------------------------------------------------------------------------------------------
7SAP S. argenteus            --------------------------------------------------------------------------------------------------------------
```

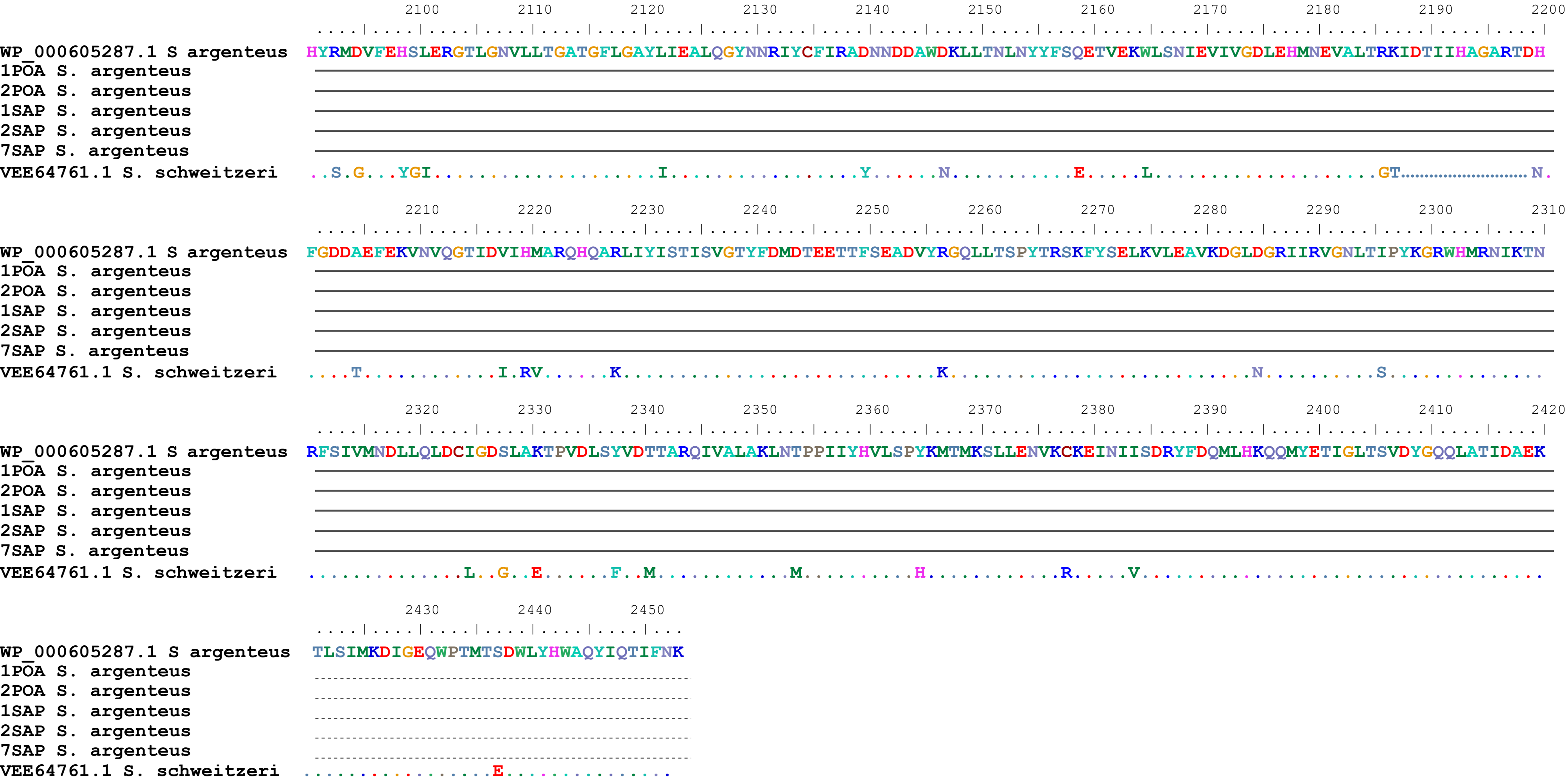

Supplemental Figure 1. Alignment of partial amino acid sequences of NRPS from five Brazilian isolates, *S. argenteus* and *S. schweitzeri* type strains.
